# Assessing the Impact of Female Genital Mutilation/Cutting on Genital Inflammation and Microbiota Among Kenyan Female Sex Workers

**DOI:** 10.1111/aji.70250

**Published:** 2026-05-04

**Authors:** James Pollock, Rachel Liu, Elizabeth Rwenji, Evelyne Orobi, Suji Udayakumar, Sanja Huibner, Mary Kung'u, Rhoda Kabuti, Hellen Babu, Maisha Fiti Study Champions, Erastus Irungu, Pauline Ngurukiri, Peter Muthoga, Wendy Adhiambo, Helen A. Weiss, Janet Seeley, Tanya Abramsky, Joshua Kimani, Tara S. Beattie, Rupert Kaul

**Affiliations:** ^1^ Department of Immunology University of Toronto Toronto Canada; ^2^ Department of Medicine University of Toronto Toronto Canada; ^3^ Partners For Health and Development in Africa (PHDA) UNITID, College of Health Sciences Nairobi Kenya; ^4^ MRC International Statistics and Epidemiology Group, Department of Infectious Disease Epidemiology London School of Hygiene and Tropical Medicine London UK; ^5^ Department of Global Health and Development London School of Hygiene and Tropical Medicine London UK

**Keywords:** female genital mutilation/cutting, female sex workers, genital inflammation, HIV susceptibility, kenya, vaginal microbiome

## Abstract

**Problem:**

Female genital mutilation/cutting (FGM/C) is harmful to physical, mental, and reproductive health, though the effect of this practice on a woman's HIV susceptibility is poorly understood. Despite the known associations of FGM/C with short‐term vaginal epithelial damage, neither genital inflammation nor the genital microbiome have been explored in women who have undergone FGM/C. In this study we compare the genital immune milieu and microbiome among female sex workers (FSWs) by FGM/C status, hypothesizing that these biological factors are dysregulated in women who have undergone FGM/C, heightening their risk of HIV acquisition.

**Method of Study:**

1003 FSWs in Nairobi, Kenya, were enrolled in the Maisha Fiti study and visited a study clinic up to three times from June 2019 to March 2021. Participants self‐reported any previous exposure to FGM/C as well as other relevant sociodemographic factors. Levels of proinflammatory cytokines and soluble E‐cadherin (sE‐cad), a biomarker of epithelial barrier disruption, were measured by multiplex immunoassay using self‐collected cervicovaginal secretion samples provided by HIV‐uninfected participants. Genital inflammation was defined using a composite score of inflammatory cytokines previously associated with HIV acquisition. The presence of inflammation was compared longitudinally between groups using mixed models to control for potential confounders including age, bacterial vaginosis (BV) status as defined by Nugent score, and others. Vaginal bacterial abundance, Shannon diversity, and total levels of key vaginal bacteria were measured by qPCR and compared by FGM/C status in an exploratory analysis.

**Results:**

44 of 1003 (4%) participants had undergone Type I or II FGM/C. These participants were older (*p* < 0.001) and more likely to test positive for herpes simplex virus‐2 (HSV‐2; *p* = 0.04), and less likely to have completed primary education (*p* = 0.03). Among HIV‐uninfected participants, there was no evidence that genital inflammation was associated with FGM/C status after controlling for potential confounders (aOR = 0.70; 95% CI: 0.31–1.59; *p* = 0.40). There was no evidence of a difference in BV prevalence (*p* > 0.99), total bacterial abundance (*p* = 0.96), or Shannon diversity (*p* = 0.15) by FGM/C status.

**Conclusions:**

Type I or II FGM/C was not associated with genital inflammation or microbial dysregulation in the long‐term among HIV‐negative FSWs in this cohort. This may be due to the duration elapsed since FGM/C occurred or the lowered mucosal immune activation previously observed in FSWs.

## 1 Introduction

Female genital mutilation/cutting (FGM/C) is defined by the WHO as the intentional partial or complete removal of external female genitalia for nonmedical reasons [1]. FGM/C is traditionally practiced in West Africa but also in countries in Europe [2], the Middle East [3], Latin America [4], Southeast Asia [5], and in diaspora communities around the world [6]. While FGM/C is usually performed for cultural purposes, many consider FGM/C to be a form of systemic gender‐based violence (GBV) [7, 8, 9]. Globally, 200 million women and girls have undergone FGM/C, and 4 million women and girls annually are at risk of undergoing the practice [1]. Although criminalization efforts have caused a decline in FGM/C practices, the WHO estimates that absolute incidence will increase due to the rising medicalization of the practice and limited success in outreach efforts [10].

FGM/C is classified into four types: Type I, the removal of the prepuce, with total or partial removal of the clitoris; Type II, the removal of the clitoris with partial or total removal of the labia minora; Type III, infibulation, or the removal of all external genitalia with stitching/narrowing of the vaginal introitus; and Type IV, which includes other forms of FGM/C such as pricking, piercing, or stretching of the clitoris or labia minora, introducing corrosive substances or herbs to the vagina for the purposes of tightening it, cauterization, or any other procedure that falls under the definition of FGM/C [1, 11]. FGM/C can cause medical complications, including septicemia, hemorrhagic shock, tetanus, reduced fertility, vesicovaginal fistulae, preterm delivery, mental health issues, and death [12]. Women who have undergone FGM/C experience reduced sexual function, with most studies finding diminished sexual desire, arousal, lubrication, orgasm, and satisfaction compared to controls, possibly in a dose‐response relationship, since women with a more severe excision (type III FGM/C) report worse sexual function relative to women with type I FGM/C [13, 14, 15, 16].

It has been hypothesized that FGM/C influences HIV susceptibility through behavioral mechanisms, such as alterations in sexual practices, and/or biological mechanisms, such as local changes to the genital microenvironment [17], however few studies have assessed this. A recent systematic review of the literature identified 14 quantitative studies on HIV and FGM/C that were of moderate quality, had small sample sizes and did not adjust for confounders, leading to inconclusive findings regarding the presence and directionality of an association between FGM/C and HIV risk [18].

Two important drivers of biological HIV risk, genital inflammation and the vaginal microbiota, have not been evaluated among women with FGM/C despite the plausible effect of FGM/C on these factors. First, the mechanical disruption of the epithelium usually associated with the FGM/C procedure would be expected to elicit a robust inflammatory and wound‐healing response in the short/medium term. The FGM/C procedure has been associated with infection at the incision sites through instrument contamination [19] which exacerbates this immune response. Second, alterations to vaginal tissue structure may change the oxygen or nutrient availability in the genital microenvironment, which could in turn alter the growth of bacteria previously linked to an increased or decreased risk of HIV acquisition [20]. Infibulation and reinfibulation (Type III FGM/C) specifically could cause drastic and ongoing alterations to oxygen tension, disrupting lactobacilli dominance which is generally HIV‐protective [21]. A 2001 study of health outcomes among Gambian women [22] found an association between FGM/C status and bacterial vaginosis (BV), an indicator of increased bacterial abundance and diversity that is independently associated with HIV acquisition [23], however this finding has not since been replicated.

We hypothesized that prior experience of FGM/C is associated with increased vaginal proinflammatory cytokine concentrations and bacterial abundance and diversity compared to women without experience of FGM/C. Further, we hypothesized that FGM/C experience is associated with an increase in soluble E‐cadherin (sE‐cad), a marker of epithelial barrier disruption. In this prospective cohort study, we assessed genital inflammation and indicators of microbial abundance and diversity by FGM/C status among Kenyan female sex workers (FSWs).

## 2 Materials and Methods

### 2.1 Study Design and Setting

This prospective cohort study included participants from the Maisha Fiti study, which has been described previously [24, 25]. All participants provided written formal consent. The Maisha Fiti study was approved by the Kenyatta National Hospital Ethics and Research Committee (KNH ERC P778/11/2018), the London School of Hygiene and Tropical Medicine (LSHTM) Ethics Committee (Approval number: 16229) and the University of Toronto ethics committee (Approval number: 37046).

The Maisha Fiti study was a longitudinal mixed‐method study conducted from June 2019‐March 2021 that investigated the effect of GBV on genital inflammation among FSWs. Participants were enrolled from seven sex worker outreach program (SWOP) clinics across Nairobi and visited the study clinic up to three times, approximately 6 months apart.

### 2.2 Study Participants

Participants were randomly selected from all SWOP clinic attendees who had accessed SWOP services in the past 12 months, were aged 18–45 years, and did not have an underlying chronic illness (other than HIV) that was likely to alter host immunology.

**FIGURE 1:**
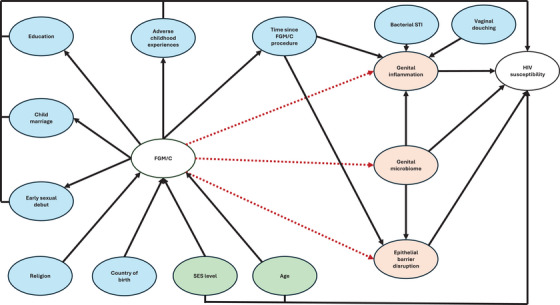
Conceptual framework pathways of plausible associations between FGM/C and biological HIV susceptibility. Effect modifiers (blue) may change the overall effect of FGM/C on biological HIV susceptibility, based on their presence or absence. Age and SES level (green) could plausibly confound the association between FGM/C and biological HIV susceptibility. This study focuses on the effect of FGM/C on genital inflammation, the genital microbiome, and epithelial barrier disruption (red). Abbreviation: SES, socioeconomic status; FGM/C, female genital mutilation/cutting; HIV, human immunodeficiency virus.

Participants were asked to complete a behavioral survey to collect sociodemographic data. One of the questions, “Have you ever had a surgical procedure used for modifying the vagina, or restoration of the hymen; including female genital circumcision, incision with insertion of substance into the lesion (scarification process, tattoos of the vulva or labia)?” was felt by the study team to lack sufficient specificity to be able to discern FGM/C from other experiences, and the FGM/C type that participants had undergone. To remedy this, we produced an additional short questionnaire to clarify the responses of the participants who answered “Yes” to this question. Participants that indicated possible FGM/C exposure in the original survey were recalled by telephone in June 2021 to be interviewed using the new questionnaire (Figure 1). The exposure variable for this analysis was FGM/C status, as indicated by the survey question, “Have you ever undergone female genital cutting?”.

Blood samples were collected from all participants for HIV diagnostics, and blood from HIV‐uninfected participants was further tested for HSV‐2 infection. Urine was collected for *Chlamydia trachomatis* and *Neisseria gonorrhoeae* diagnostics, and pregnancy testing. Participants provided self‐collected vaginal swab samples for BV assessment via Nugent score and for further microbiome sequencing, as well as self‐collected menstrual cup samples for immune assays.

### 2.3 Biological Assays

Genital secretion samples obtained from self‐collected menstrual cups were processed (Supplementary Appendix 1) and frozen at −80 C. In Toronto, samples from HIV‐uninfected participants were thawed and centrifuged at 500 g for 5 min. Supernatant was collected and used for soluble immune factor quantification by multiplex electrochemiluminescent assay (MSD; Meso Scale Discovery). Continuous levels of Interleukin (IL)‐1*α*, IL‐1*β*, IL‐6, IL‐8, interferon gamma‐induced protein (IP)‐10, monocyte chemoattractant protein (MCP)‐1, monokine induced by gamma interferon (MIG), macrophage inflammatory protein (MIP)‐1*α*, MIP‐1*β*, MIP‐3*α*, tumour necrosis factor (TNF), soluble E‐cadherin (sE‐cad), and matrix metallopeptidase (MMP)‐9 were measured in duplicate using the multiplex platform.

DNA from the genital secretion pellets of HIV‐uninfected participants was extracted (DNase PowerSoil Pro kits; Qiagen) and analyzed through 16S rRNA sequencing via Illumina MiSeq (Table S1). In addition, TaqMan‐based RT‐qPCR was used to assess absolute abundance of 16S rRNA, in order to calculate the estimated absolute abundance of vaginal species, as well as to directly measure the absolute abundance of key vaginal species *Lactobacillus crispatus*, *Lactobacillus iners*, *Prevotella bivia* and *Gardnerella vaginalis*.

### 2.4 Statistical Analysis

**FIGURE 2:**
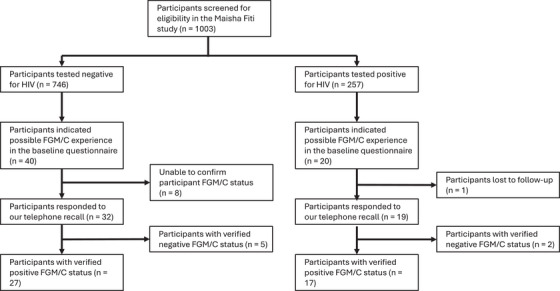
Flowchart of participant HIV screening and FGM/C status verification through telephone recall. Abbreviation: FGM/C, female genital mutilation/cutting.

Sociodemographic factors assessed by the baseline study questionnaire were explored in a descriptive analysis using chi‐square (*χ* ^2^) tests, Fisher's exact tests, and Welch's *t*‐tests to compare sociodemographic factors between participants who had undergone FGM/C and those who had not. Since the focus of this study was biological HIV susceptibility, participants living with HIV were omitted from subsequent analyses. The primary outcomes for this study were genital inflammation, defined by a composite score of nine proinflammatory cytokines (Supplementary Appendix 1), and sE‐cad, a marker of epithelial barrier disruption. Secondary outcomes included BV status, Shannon index, and levels of 16S rRNA, *L. crispatus*, *L. iners*, *P. bivia* and *G. vaginalis*. Logistic mixed‐effects regression models were constructed to explore the direct association between presence/absence of genital inflammation and self‐reported FGM/C status adjusting for potential confounders based on their known or plausible association with genital inflammation (Figure 2). These models were adjusted for age, socioeconomic status (SES), BV status, vaginal cleaning practices using soap, bacterial STI prevalence, and high risk substance use as defined by the WHO ASSIST tool. Data from all three visits were included in these models, and participant ID was included as a random effect to allow for the inclusion of correlated observations at all three time points without violating the assumption of independence. Additionally, to understand the mediation effect of BV status, we constructed a mixed effects model of genital inflammation and FGM/C status that controlled for the same variables except BV status.

The effect of FGM/C on log‐transformed continuous sE‐cad levels, the other co‐primary study endpoint, was estimated using mixed effects linear regression, using Kenward‐Roger approximation from the R package lmerTest (v. 3.1‐3) to assess the significance of FGM/C as a fixed effect. We performed Welch's *t*‐tests to understand the individual cross‐sectional associations between FGM/C status and each log‐transformed immune biomarker outcome at the baseline visit as exploratory secondary endpoints.

**FIGURE 3:**
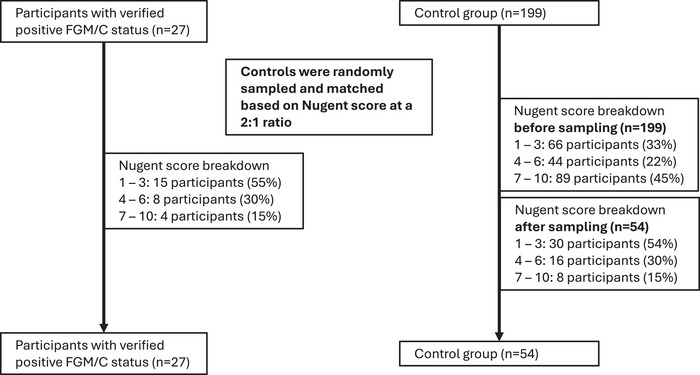
Flowchart of participant selection for microbiome comparisons. Abbreviation: FGM/C, female genital‐mutilation/cutting.

Along with pellet samples from participants who had undergone FGM/C, control samples from participants who had not undergone FGM/C were selected for bacterial sequencing and qPCR. As this selection of control samples was enriched for BV prevalence for the purposes of a separate study, control samples were randomly sampled and matched to case samples by Nugent score at a 2:1 ratio to be able to make appropriate comparisons between the groups for further exploratory microbiome analyses (Figure 3). Total bacterial abundance, Shannon diversity index, and continuous levels of key vaginal bacteria were compared by FGM/C status using Welch's *t*‐test.

Principal component analysis (PCA) was used to explore patterns in the cytokine and bacterial data. Eigenvalues were calculated by parallel analysis, and principal components (PCs) with eigenvalues greater than those from simulated data at the 95% percentile level were selected. PC unit vectors of levels of the 13 soluble immune factors, 16S rRNA, *L. crispatus*, *L*. *iners*, *G. vaginalis*, and *P. bivia* were projected onto PC1 versus PC2 space. Correlations between individual PC scores versus cytokine and bacterial data were evaluated. All analyses were performed in R (v. 4.3.1) and Prism 10 (v. 10.3.1).

## 3 Results

### 3.1 Participant Characteristics

In total, 44 (4%) of 1003 Maisha Fiti participants reported having undergone FGM/C (Table 1). Of these, 34 (78%) reported Type I FGM/C, 8 (18%) reported Type II FGM/C, and 2 (5%) were unsure about the type of FGM/C they had undergone. No participants underwent FGM/C during the study follow‐up period, and no participants had experienced Type III or IV FGM/C. Due to low heterogeneity in self‐reported FGM/C type among HIV‐uninfected participants, we did not stratify by FGM/C type in this analysis. A median 24 years (range 2–35 years) had elapsed since each participant's initial FGM/C procedure. The median age at the time of the FGM/C procedure was 14 years (range 5–25 years), and only 10 (23%) women had given consent to undergo FGM/C.

**TABLE 1:** Participant characteristics at the time of FGM/C procedure and at the time of the baseline study visit.

Characteristics	No self‐reported FGM/C (*n* = 959)	Self‐reported FGM/C (*n* = 44)	*p* value
DISTAL FACTORS			
Self‐reported Type I FGM/C	−	34 (77%)	−
Self‐reported Type II FGM/C	−	8 (18%)	−
Self‐reported FGM/C, unsure about type	−	2 (5%)	−
Age at FGM/C exposure (median, range)	−	14 (5–25)	−
Consented to FGM/C	−	10 (23%)	−
Age at sexual debut (median, range)	16 (2–26)	16 (5–26)	0.51
*Country of birth*			0.65
Kenya	945 (99%)	44 (100%)	
Not Kenya	14 (1%)	0 (0%)	
*Religion*			0.93
Catholic	358 (37%)	17 (39%)	
Protestant	511 (53%)	23 (52%)	
Muslim	43 (4%)	3 (7%)	
No religion	34 (4%)		1 (2%)
Other	11 (1%)	0 (0%)	
Decline to respond	2 (<1%)	0 (0%)	
*Socioeconomic status*			0.31
Lower	320 (33%)	14 (32%)	
Middle	323 (34%)	11 (25%)	
Upper	314 (33%)	19 (43%)	
*Education*			**0.04**
Did not complete primary	157 (16%)	12 (27%)	
Primary	500 (52%)	25 (57%)	
Secondary or higher	302 (31%)	7 (16%)	
*Number of adverse childhood experiences*			0.85
0–4	270 (28%)	12 (27%)	
5–8	525 (55%)	23 (52%)	
9–12	164 (17%)	9 (20%)	
PROXIMAL FACTORS			
Age at time of baseline study visit (median, range)	32 (18–45)	38.5 (20–45)	**<0.001**
Living with HIV	240 (25%)	17 (39%)	0.06
Any mental health problem^*^	230 (24%)	13 (30%)	0.51
Number of partners, past 7 days (median, range)	3 (0–70)	3 (0–30)	0.60
Vaginal washing	561 (58%)	26 (59%)	>0.99
Bacterial vaginosis	126 (17%)	4 (15%)	>0.99
*Laboratory‐confirmed STIs*			
*Chlamydia trachomatis*	65 (7%)	2 (5%)	0.67
*Neisseria gonorrhoeae*	27 (3%)	0 (0%)	0.63
*Trichomonas vaginalis*	29 (3%)	2 (5%)	0.64
HSV‐2^**^	362 (50%)	19 (70%)	**0.04**

^*^Depression, anxiety, or post‐traumatic stress disorder

^**^HSV‐2 serology was only completed for HIV‐uninfected participants

At the baseline study visit, participants who had undergone FGM/C were older (median age 38.5 vs 30, *p* < 0.001) and less likely to have completed secondary or higher education (16% vs 31%, *p* = 0.03) than those who had not undergone FGM/C. While not statistically significant, HIV prevalence was higher among women who had undergone FGM/C (39% vs 25%, *p* = 0.06). There was no evidence of a difference in reported age at sexual debut (median age 16 vs 16 years, *p* = 0.51) or in the reported number of male sexual partners in the past 7 days (median 3 vs 3, *p* = 0.60). Among HIV‐uninfected participants, women who had undergone FGM/C were more likely to test positive for HSV‐2 (70% vs 50%, *p* = 0.04).

### 3.2 Genital Inflammation

**FIGURE 4:**
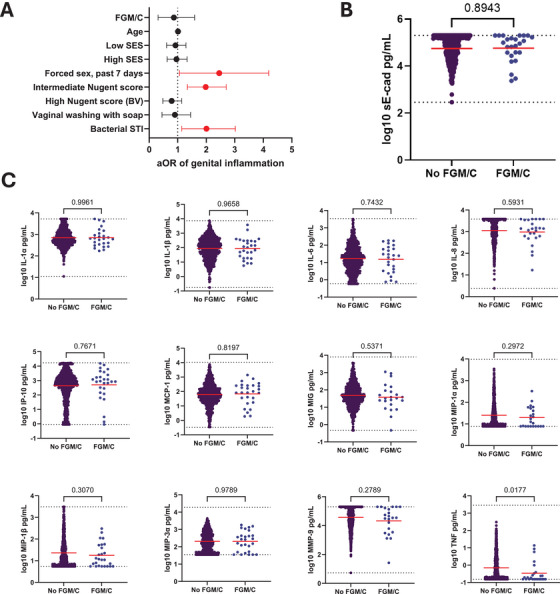
**(A**) Forest plot of the effect of FGM/C status on genital inflammation in a mixed effects model. SES, socioeconomic status; BV, bacterial vaginosis; aOR, adjusted odds ratio. (**B**) Baseline levels of sE‐cad among HIV participants by FGM/C status. Groups were compared by Welch's *t*‐test. Upper and lower limits of detection are indicated by dashed lines. (**C**) Baseline levels of proinflammatory cytokines among HIV‐uninfected participants by FGM/C status. Groups were compared by Welch's *t*‐test. Upper and lower limits of detection are indicated by dashed lines.

Cytokine data were available for 711/746 (95%) HIV‐uninfected participants at baseline, of whom 351 (49%) participants had vaginal inflammation. Cytokine data were available for 26/27 participants who had undergone FGM/C, of whom 11 (42%) had genital inflammation at baseline, compared to 340 (50%) participants who had not undergone FGM/C. In our longitudinal analysis, there was no evidence that FGM/C status was associated with subsequent genital inflammation (aOR = 0.70, 95% CI: 0.31–1.59; *p* = 0.40), adjusted for age, SES, forced sex experience in the seven days prior to the study visit, BV status, vaginal cleaning practices using soap, bacterial STI prevalence, and high‐risk substance use (Figure 4A; Table 2). In this model, recent forced sex experience (aOR = 2.11, 95% CI: 1.06–4.19; *p* = 0.03), intermediate Nugent score (aOR = 1.90, 95% CI: 1.34–2.70; *p* < 0.001) and bacterial STI prevalence (aOR = 1.86, 95% CI: 1.14–3.02; *p* = 0.01) were associated with genital inflammation. Our model that did not adjust for BV status produced a similar output (Table S2). There was also no evidence that sE‐cad levels were associated with FGM/C status (adjusted mean difference (aMD) = 0.06, *p* = 0.31; Figure 4B). Participants that had undergone FGM/C had lower levels of vaginal TNF (mean −0.46 vs −0.15 pg/mL; *p* = 0.01), while there was no evidence of differences in log‐transformed levels of IL‐1*α*, IL‐1*ß*, IL‐6, IL‐8, MCP‐1, IP‐10, MIP‐1*α*, MIP‐1*ß*, MIP‐3*α*, or MMP‐9, compared to women who had not undergone FGM/C (Figure 4C).

**TABLE 2:** Associations between genital inflammation and FGM/C experience (mixed‐effects logistic regression model).

	Fixed effect estimate	aOR	95% CI	*p* value
FGM/C	−0.36	0.70	0.31–1.59	0.39
Age	0.006	1.01	0.99–1.03	0.56
Medium SES	0 (reference)			
Low SES	−0.11	0.90	0.61–1.31	0.57
High SES	−0.08	0.92	0.63–1.34	0.66
Forced vaginal sex, past 7 days	0.74	2.09	1.06–4.16	**0.03**
Low nugent score	0 (reference)			
Intermediate nugent score	0.65	1.91	1.35–2.71	**<0.001**
High nugent score	−0.30	0.74	0.48–1.14	0.17
Vaginal cleaning (soap)	−0.22	0.80	0.44–1.44	0.46
Bacterial STI^*^	0.61	1.84	1.13–3.00	**0.01**

Bold *p* values denote significance at the 5% level.

^*^Chlamydia, gonorrhea or syphilis infection.

Abbreviation: aOR, adjusted odds ratio; SES, socioeconomic status; STI, sexually transmitted infection.

### 3.3 Vaginal Microbiome

**FIGURE 5:**
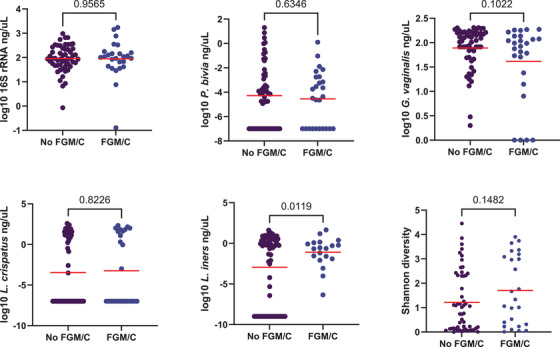
Impact of FGM/C status on key indicators of vaginal bacterial abundance and diversity. Groups were compared by Welch's t‐test.

Gram staining was performed using vaginal swabs provided by Maisha Fiti participants at each study visit. 140 participants met Nugent score criteria for BV at the baseline study visit, indicating higher total bacterial density and diversity among these participants. BV prevalence was not significantly different among participants who had undergone FGM/C compared to those who had not (Table 1; 17% vs 15%, *n* = 1003, *p* > 0.99). Then, among those selected for further microbiome sequencing, we found that estimated absolute bacterial abundance (*t* = 0.05, *p* = 0.96) and Shannon diversity (*t* = −1.47, *p* = 0.15) did not differ by FGM/C status (Figure 5). Similarly, there was no difference in levels of the key vaginal species *P. bivia* (*t* = 0.48, *p* = 0.63), *G. vaginalis* (*t* = 1.68, *p* = 0.10), or *L. crispatus* (*t* = −0.23, *p* = 0.82). Levels of *L. iners* were significantly higher among those that had undergone FGM/C (*t* = −2.58, *p* = 0.01).

### 3.4 Principal Component Analysis

**FIGURE 6:**
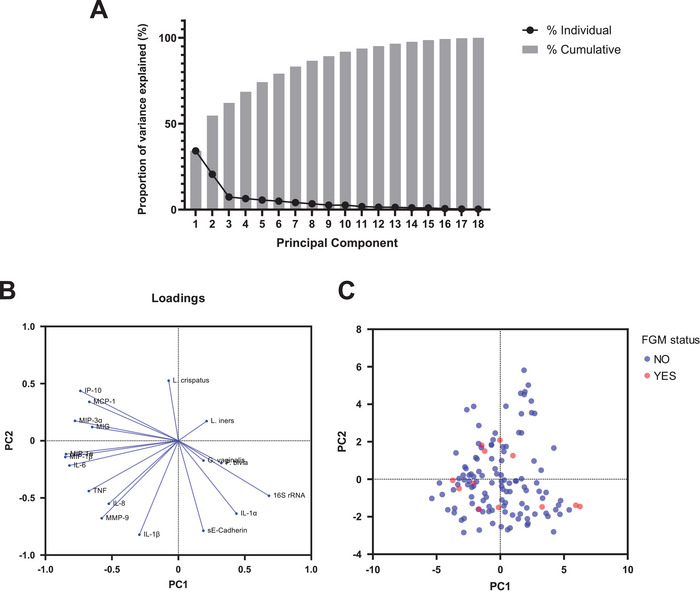
The PCA of soluble immune factors, total bacterial load, and individual bacterial species. (**A**) Proportion of variance plot. (**B**) Loadings plot. (**C**) PC scores plot.

Complete immune and microbiome data were available for 126 participants, who were included in the PCA. Of the 18 PCs generated, PC1 and PC2 accounted for 54.7% of the total variance of the immune and bacterial data (Figure 6A). PC1 was positively correlated with levels of 16S rRNA (*R* = 0.68), IL‐1*α* (*R* = 0.43), and *P. bivia* (*R* = 0.32), and negatively correlated with levels of MIP‐1*β* (*R* = −0.85), MIP‐1*α* (*R* = −0.85), and IL‐6 (*R* = −0.82) (Figure 6B). PC2 was positively correlated with levels of *L. crispatus* (*R* = 0.52), IP‐10 (*R* = 0.43), and MCP‐1 (*R* = 0.34), and negatively correlated with levels of IL‐1*β* (*R* = −0.82), sE‐cad (*R* = −0.79), and MMP‐9 (*R* = −0.68). When PC scores from the data were plotted along PC1 and PC2 axes and annotated by FGM/C status, no visually distinct clusters were observed (Figure 6C).

## 4 Discussion

In this study, we found no evidence of differences in genital inflammation between women who had and had not undergone Type I or II FGM/C. Levels of sE‐cad, a marker of epithelial barrier disruption, and BV prevalence, an indicator of increased bacterial abundance and diversity. were not associated with FGM/C status. In an exploratory analysis of soluble immune factors, TNF concentrations were significantly lower among women who had undergone FGM/C. FGM/C experience was associated with increased age and positive HSV‐2 serostatus, and negatively associated with educational outcomes among these women. While not statistically significant, we observed a positive trend between FGM/C experience and HIV status.

Global data on the sociodemographic characteristics of women who have undergone FGM/C are almost as heterogeneous as the practice of FGM/C itself; however, our findings are broadly reflective of previous research conducted among Kenyan women who have undergone FGM/C. The prevalence of FGM/C in this cohort of FSWs in Nairobi was 4%, in line with previous studies conducted in Kenya that stratify by region and urban versus rural place of residence [26, 27, 28]. As with previous research, we found associations between FGM/C experience and lower levels of education [27, 28, 29, 30], as well as older age [27, 28, 29] and HSV‐2 serostatus [31], though age was not independently associated with FGM/C experience when age and HSV‐2 serostatus were included in the same model (data not shown). While the methodology used to assess SES can differ between studies, many find associations between FGM/C experience and low levels of wealth [29, 32, 33]. We did not observe an association between religion and FGM/C status in this cohort. Some previous studies have found that Muslim women are more likely to have undergone FGM/C [34], something that we could not assess robustly due to a relatively low frequency of Muslim participants in our study.

It has been previously hypothesized [35] that the extensive trauma and damage caused to the vaginal epithelium by FGM/C procedures would translate into increased immune cell recruitment and cytokine production at the site of the wound, representing a biological pathway increasing HIV risk. We did not observe higher concentrations of pro‐inflammatory cytokines among women who had undergone FGM/C in this study, and indeed in our exploratory analysis of individual cytokines, women who had undergone FGM/C had significantly lower levels of TNF, an important activator of several inflammatory signaling pathways, which could reflect modest mucosal quiescence. However, it seems likely that any immediate immune changes would be relatively short‐lived, since previous studies have shown that inflammation resulting from genital trauma does not persist for more than a short period. Specifically, in a study of heterosexual couples in Toronto, Canada, we found that vaginal inflammatory cytokines rapidly increased after penile‐vaginal sex and returned to baseline within 72 h [36]. In this same cohort, genital epithelial trauma caused by cytobrush sampling induced a brief increase in pro‐inflammatory cytokines that dissipated within 48 h after sampling [37]. In a previous analysis of the Maisha Fiti cohort, we found that women who were forced to have vaginal sex within the past 7 days were more likely to have genital inflammation, whereas women who were exposed to physical or sexual violence over a longer time frame (within the past 6 months) did not [38]. Since women in the current Nairobi cohort who had experienced FGM/C were of median age 35 at the time of the study and had undergone FGM/C at median age 14, we are not able to comment on inflammation that may well be induced by the FGM/C procedure. However, our results demonstrate that FGM/C (at least of the type prevalent within our cohort, see below) was not associated with long‐lasting inflammatory effects that persisted into adulthood.

Nearly all participants who self‐reported FGM/C described this experience as type I FGM/C, which was not associated with long‐term inflammatory effects or microbiota alterations. While beyond the scope of our study, we hypothesize that type III FGM/C, the infibulation of the vaginal introitus, may alter the vaginal microenvironment and skew towards a more anaerobic, BV‐type vaginal microbiota that has been demonstrated in multiple prior studies to be inflammatory. Reinfibulation, performed after childbirth or gynecological procedures, may increase the persistence of the inflammatory environment for years after the initial FGM/C procedure. Therefore more research is required before we can confidently state that FGM/C is not associated with genital inflammation.

Future studies should explore the effect of FGM/C on adolescent girls in regions where FGM/C is commonly practiced and assess longitudinally whether FGM/C causes disruptions to the genital immune microenvironment in the days, weeks, and months after they undergo FGM/C. Additionally, since previous studies have observed lower mucosal immune activation among sex workers compared to non‐sex workers [39], future studies should also consider enrolling non‐FSWs to address these research questions.

To our knowledge, this is the first study of genital cytokines and microflora in women in sub‐Saharan Africa to collect data on and stratify by FGM/C status. We used a holistic definition of genital inflammation that has been previously associated with HIV seroconversion, and collected next‐generation sequencing data for the genital bacterial consortia of all women who reported FGM/C experience in this study. Our multivariate statistical approach permitted us to investigate the effect of FGM/C on genital inflammation longitudinally, and while controlling for potential confounders that could obscure the true effect of FGM/C exposure on our primary endpoint. Our study does not come without limitations, though. As reflected in the relatively wide confidence intervals reported in our primary analysis, we may have been underpowered to detect significant differences in genital inflammation between groups due to the relatively low number of participants who had experienced FGM/C compared to those who had not. As mentioned, we were unable to assess the biological impact of Type III FGM/C, which may cause more profound alterations to genital immunology than the Type I and II procedures reported in this study. FGM/C status and type were self‐reported, and since two participants were unsure which type of FGM/C they had experienced, we cannot eliminate the possibility of misclassification bias in this study. We did not collect data on ethnicity, which has been previously associated with FGM/C practices. The matched pairs design of the microbiome analysis allows us to compare microbial parameters independent of BV status, however these findings may not be generalizable outside of BV‐matched strata. Lastly, we were unable to assess HIV incidence as an outcome, since no seroconversions were observed in the Maisha Fiti study.

Our results suggest that effects of Type I or II FGM/C on the risk of HIV acquisition are more likely to be driven by sociobehavioral rather than biological pathways. However, the drastic alteration of genital anatomy caused by FGM/C practices, particularly type III FGM/C, means that further investigation into how these procedures affect HIV susceptibility is warranted.

## Funding

The Maisha Fiti Study is funded by the Medical Research Council MRC and the UK Department of International Development (DFID) (MR/R023182/1) under the MRC/DFID Concordat agreement. HW is supported by the MRC and the DFID under the MRC/DFID Concordat agreement and is also part of the EDCTP2 programme supported by the European Union. RK is supported by the Canadian Institute of Health Research (CIHR) grant #PJT‐180629 and #PJT‐156123.

## Ethics Statement

The Maisha Fiti study was approved by the Kenyatta National Hospital Ethics and Research Committee (KNH ERC P778/11/2018), the London School of Hygiene and Tropical Medicine (LSHTM) Ethics Committee (Approval number: 16229) and the University of Toronto ethics committee (Approval number: 37046).

## Conflicts of Interest

The authors declare no conflicts of interest.

## Supporting information




**Supplementary Table 1**. Baseline sociodemographic characteristics among HIV‐uninfected participants.

**Supplementary Table 2**. Associations between genital inflammation and FGM/C experience, without adjustment for bacterial vaginosis (mixed‐effects logistic regression model).

Supplementary Appendix 1

## Data Availability

The data that support the findings of this study are available on request from the corresponding author. The data are not publicly available due to privacy or ethical restrictions.
