# Supplementary material for: Assessing the Impact of Female Genital Mutilation/Cutting on Genital Inflammation and Microbiota Among Kenyan Female Sex Workers
Source: Am J Reprod Immunol. 2026 May 4;95:e70250. doi: 10.1111/aji.70250 (PMC13138364; doi:10.1111/aji.70250)
Supplement: Supplementary file 1 — Supplementary Table 1. Baseline sociodemographic characteristics among HIV‐uninfected participants. [file AJI-95-e70250-s001.docx]

**Supplementary Table 1. Baseline sociodemographic characteristics among HIV-uninfected participants.**

| Characteristics | No self-reported FGM/C (n = 719) | Self-reported FGM/C (n = 27) | p value |
| --- | --- | --- | --- |
| DISTAL FACTORS |  |  |  |
| Self-reported Type I FGM/C | -- | 25 (93%) | -- |
| Self-reported Type II FGM/C | -- | 2 (7%) | -- |
| Age at FGM/C exposure (median, range) | -- | 14 (5 - 25) | -- |
| Consented to FGM/C | -- | 5 (19%) | -- |
| Age at sexual debut (median, range) | 17 (2 – 26) | 17 (13 – 26) | 0.74 |
| *Country of birth* |  |  | 0.99 |
| Kenya | 709 (99%) | 27 (100%) |  |
| Not Kenya | 10 (1%) | 0 (0%) |  |
| *Socioeconomic status* |  |  | 0.28 |
| Lower | 239 (33%) | 10 (37%) |  |
| Middle | 235 (33%) | 5 (19%) |  |
| Upper | 245 (34%) | 12 (44%) |  |
| *Education* |  |  | 0.04 |
| Did not complete primary | 157 (16%) | 12 (27%) |  |
| Primary | 500 (52%) | 25 (57%) |  |
| Secondary or higher | 302 (31%) | 7 (16%) |  |
| *Number of adverse childhood experiences* |  |  | 0.85 |
| 0-4 | 270 (28%) | 12 (27%) |  |
| 5-8 | 525 (55%) | 23 (52%) |  |
| 9-12 | 164 (17%) | 9 (20%) |  |
| PROXIMAL FACTORS |  |  |  |
| Age at time of baseline study visit (median, range) | 30 (18-45) | 35 (20-44) | **0.03** |
| Any mental health problem* | 210 (29%) | 9 (33%) | 0.80 |
| Number of partners, past 7 days (median, range) | 3 (0-70) | 4 (0-30) | 0.52 |
| Vaginal washing | 52 (7%) | 2 (7%) | 1 |
| Bacterial STI | 92 (13%) | 2 (7%) | 0.56 |
| Bacterial vaginosis | 126 (17%) | 4 (15%) | 1 |
| HSV-2 | 362 (50%) | 19 (70%) | **0.04** |

*depression, anxiety, or post-traumatic stress disorder
