# Supplementary material for: Assessing the Impact of Female Genital Mutilation/Cutting on Genital Inflammation and Microbiota Among Kenyan Female Sex Workers
Source: Am J Reprod Immunol. 2026 May 4;95:e70250. doi: 10.1111/aji.70250 (PMC13138364; doi:10.1111/aji.70250)
Supplement: Supplementary file 2 — Supplementary Table 2. Associations between genital inflammation and FGM/C experience, without adjustment for bacterial vaginosis (mixed‐effects logistic regression model). [file AJI-95-e70250-s002.docx]

**Supplementary Table 2. Associations between genital inflammation and FGM/C experience, without adjustment for bacterial vaginosis (mixed-effects logistic regression model).**

|  | Fixed effect estimate | aOR | 95% CI | p value |
| --- | --- | --- | --- | --- |
| FGM/C | -0.38 | 0.68 | 0.30 – 1.53 | 0.35 |
| Age | 0.01 | 1.01 | 0.99 – 1.03 | 0.37 |
| Medium SES | 0 (reference) |  |  |  |
| Low SES | -0.12 | 0.89 | 0.61 – 1.29 | 0.53 |
| High SES | -0.10 | 0.90 | 0.62 – 1.30 | 0.59 |
| Forced vaginal sex, past 7 days | 0.76 | 2.13 | 1.09 – 4.18 | **0.03** |
| Vaginal cleaning (soap) | -0.08 | 0.92 | 0.52 – 1.63 | 0.78 |
| Bacterial STI* | 0.62 | 1.87 | 1.16 – 3.00 | **<0.01** |

Bold p values denote significance at the 5% level.

*Chlamydia, gonorrhea or syphilis infection.

aOR, adjusted odds ratio; SES, socioeconomic status; STI, sexually transmitted infection.
