# Supplementary material for: Assessing the Impact of Female Genital Mutilation/Cutting on Genital Inflammation and Microbiota Among Kenyan Female Sex Workers
Source: Am J Reprod Immunol. 2026 May 4;95:e70250. doi: 10.1111/aji.70250 (PMC13138364; doi:10.1111/aji.70250)
Supplement: Supplementary file 3 — Supplementary Appendix 1 [file AJI-95-e70250-s003.docx]

**Supplementary Appendix 1**

Quantification of soluble immune factors

Within two hours of each study visit, menstrual cups were transported to the Nairobi lab where they were placed in conical tubes and centrifuged at 555 g for 5 minutes. Samples were weighed and resuspended at a ten-fold dilution in sterile PBS before being centrifuged again at 1730 g for ten min. Supernatant was frozen at -80 C and transported to Toronto for soluble immune factor measurement. In Toronto, samples were thawed at room temperature and centrifuged at 500 g for 5 minutes for immune factor analysis. Interleukin (IL)-1α, IL-1β, IL-6, IL-8, interferon gamma-induced protein (IP)-10, monocyte chemoattractant protein (MCP)-1, monokine induced by gamma interferon (MIG), macrophage inflammatory protein (MIP)-1α, MIP-1β, MIP-3α, tumour necrosis factor (TNF), soluble E-cadherin (sE-cad), and matrix metallopeptidase (MMP)-9 were measured in duplicate using the MSD platform. Genital inflammation was defined by a binary composite score of inflammatory cytokines (IL-1α, IL-1ß, IL-6, IL-8, IP-10, MCP-1, MIP-1α, and MIP-1ß) that has been previously associated with HIV seroconversion. Cytokines above the median concentration threshold (based on the baseline concentration values) were considered “elevated” in a given participant. Participants with at least 5 out of 9 elevated cytokines were considered to have genital inflammation.

DNA extraction.

DNA was isolated from 250uL of CVS pellet using DNEasy PowerSoil Pro Kit (Qiagen). DNA was eluted in 60uL of the Qiagen elution buffer and analyzed through RT-qPCR and 16S rRNA amplicon sequencing.

16S rRNA gene sequencing.

Extracted CVS DNA was analyzed through 16S rRNA amplicon sequencing on the Illumina MiSeq. The V4 hypervariable region of the 16S rRNA gene was amplified using uniquely barcoded 515F (forward) and 806R (reverse) sequencing primers to allow for multiplexing. Amplification reactions were performed using 12.5 uL of KAPA2G Robust HotStart ReadyMix (KAPA Biosystems), 1.5 uL of 10 uM forward and reverse primers, 7.5 uL of sterile water and 2 uL of DNA. The V4 region was amplified by cycling the reaction at 95°C for 3 minutes, 18x cycles of 95°C for 15 seconds, 50°C for 15 seconds and 72°C for 15 seconds, followed by a 5-minute 72°C extension. All amplification reactions were analyzed duplicate to reduce amplification bias, pooled, and checked on a 1% agarose TBE gel. Pooled duplicates were quantified using PicoGreen and combined by even concentrations. The library was then purified using Ampure XP beads and loaded onto the Illumina MiSeq for sequencing, according to manufacturer instructions (Illumina, San Diego, CA). Sequencing is performed using the V2 (150bp x 2) chemistry. A single species (Pseudomonas aeruginosa DNA), a mock community (Zymo Microbial Community DNA Standard D6305), and a template-free negative control were included in the sequencing run.

Analysis of the bacterial microbiome.

The Qiime2 analysis package was used for sequence analysis, and the following functions were accessed from within the Qiime2 package: the quality of the sequencing run was first examined using FastQC and MultiQC; Cutadapt was used, following the default settings, to remove sequences with high errors rates; paired-end sequences were assembled, and quality trimmed using vsearch –fastq_mergepairs following default settings, with a –fastq_truncqual set at 2, a maxee set at 1, and minimum and maximum assemble lengths set at 250 and 255 (+2 and -3 base pairs from the expected sequence length of 253bp); assembled sequences were subjected to an additional filtering step, utilizing the quality-filter function in Qiime2; the resulting high-quality data was then processed following the deblur pipeline. Sequences were clustered into Amplicon Sequence Variant (ASV) groups and singleton sequences were removed. Taxonomy assignment was executed using the Qiime2 classify-hybrid-vsearch-sklearn function and the Average ReadyToWear trained Silva database version 138.1 ASVs with an abundance less than 0.01% are removed to reduce the potential for observing bleed-through ASVs, and ASVs identified as contaminating chloroplast or mitochondria are removed. A phylogenetic tree is created using the SEPP function available through Qiime2. The speciateIT tool was used to further annotate several OTUs to the species level. OTUs were only classified to the level compatible with Valencia (classification method for vaginal microbial communities based on composition) – as such several OTUs were only classified to the genus levels, whereas major vaginal microbiome components were classified to the species levels when able to do so. Valencia was then used to classify participants to their respective vaginal community state types (CST). Women classified as CST IV were designated as BV positive, and all other classifications were designated as BV negative.

RT-qPCR analysis.

The extracted CVS DNA was analyzed using TaqMan-based RT-qPCR through the QuantStudio 6 Flex Real-Time PCR System (Thermo Fisher Scientific) in either single-plex or multiplex assay. The protocol for quantification of *L. crispatus* and *L. iners* absolute abundance with multiplex qPCR and G. vaginalis absolute abundance single plex were adopted from previously reported assays, whereas *P. bivia* absolute abundance was quantified using primers designed using the pipeline outlined in Schneeberger et al. The total reaction volume for assays was 10 μL. Assays for *P. bivia*, *L. crispatus, L. iners*, and total bacterial abundance (16S) were performed at 95°C for 10 minutes, 45 cycles at 95°C for 15 seconds, and then at 60°C for 1 minute. Assays for G. vaginalis were performed at 95°C for 10 minutes, 45 cycles for 15 seconds, and then at 55°C for 1 minute. Data analysis was performed with QuantStudio Real-Time PCR Software version 1.3 (Applied Biosystems) and the Thermo Fisher Connect platform. Lab-grown pure cultures of bacteria and their quantified DNA (ng/mL; Qubit, Thermo Fisher Scientific) were used to create standard curves and to determine the LLOQ, defined as the lowest technical duplicates with quantifiable CT values. All values were transformed to account for sample dilutions and concentrations at/below the LLOQ were set to the LLOQ value. All values above the LLOQ were normalized to CVS sample volume and reported ng of DNA /mL of CVS.

Bacterial preparations.

*L. crispatus* (ATCC, 33820) was grown in De Man, Rogosa and Sharpe (MRS) agar, *P. bivia* was grown on TSA agar with 5% sheep’s blood (Hardy Diagnostics), *L. iners* (ATCC, 55195) were grown in New York City III agar, and *G. vaginalis* (ATCC, 14019) were grown in New York City III agar. All bacteria were grown anaerobically (80% N2; 10% CO2; 10 H2) at 37°C for 46-48hrs.
